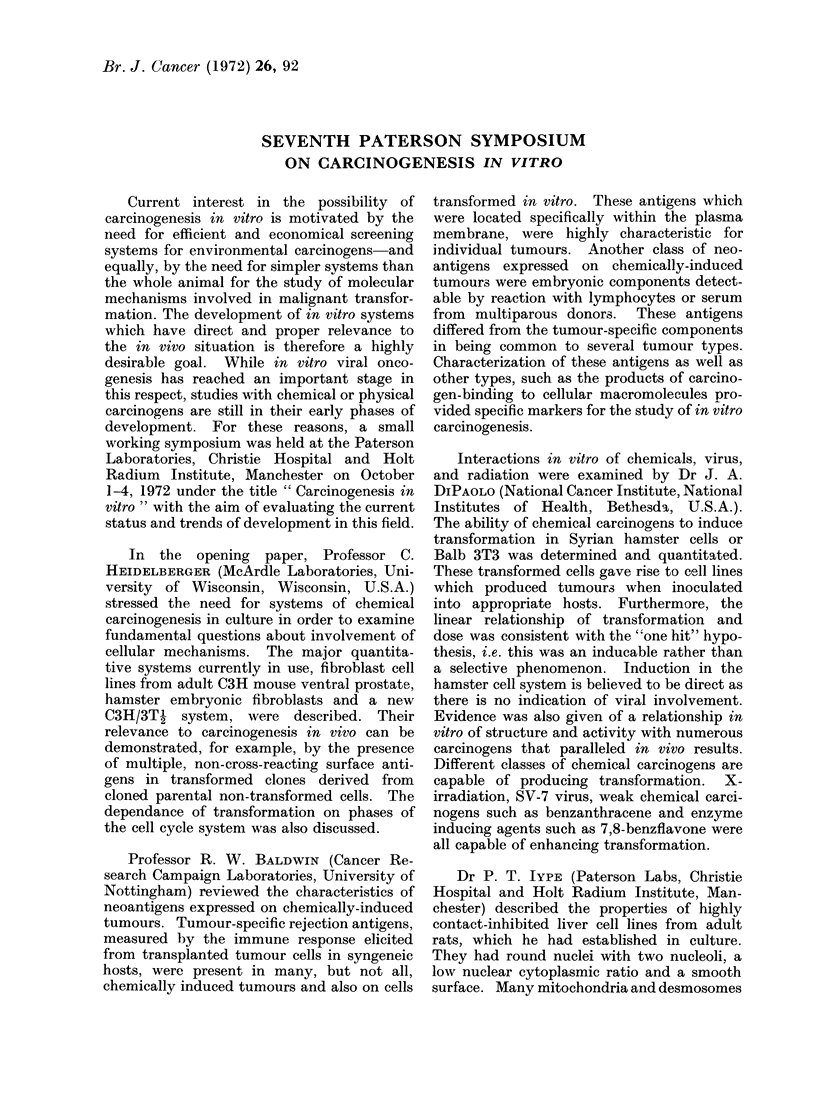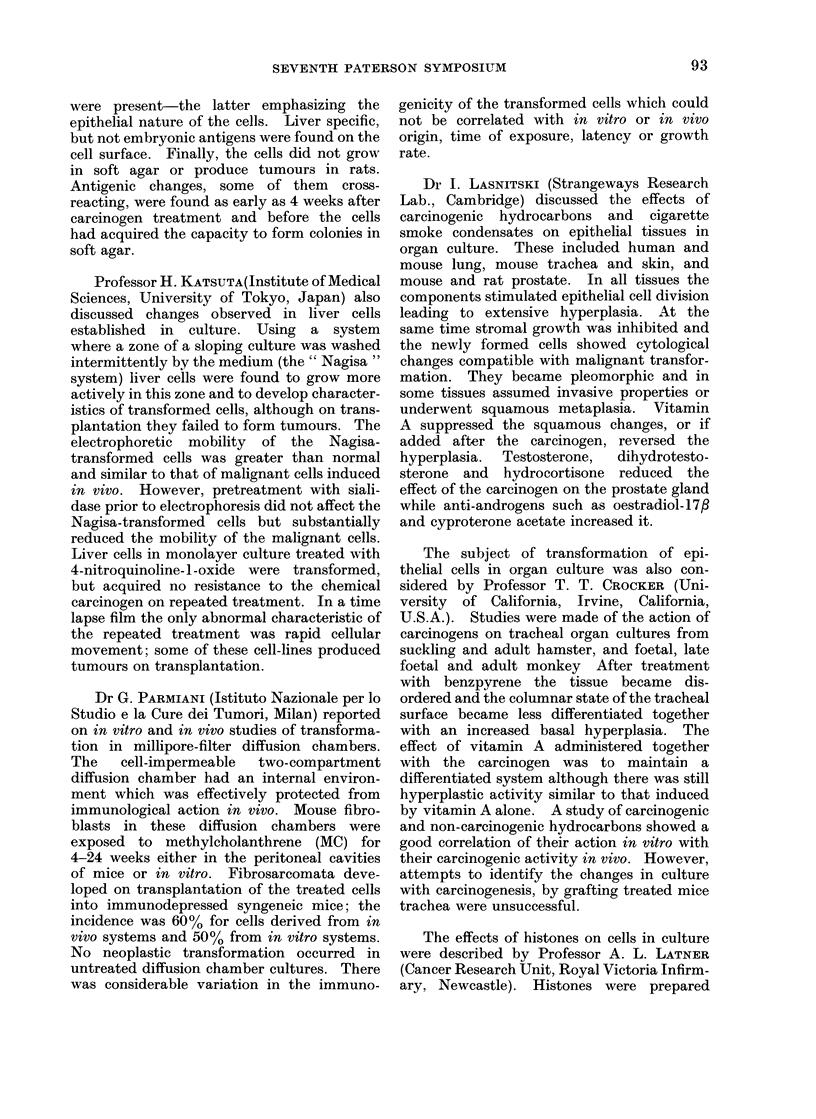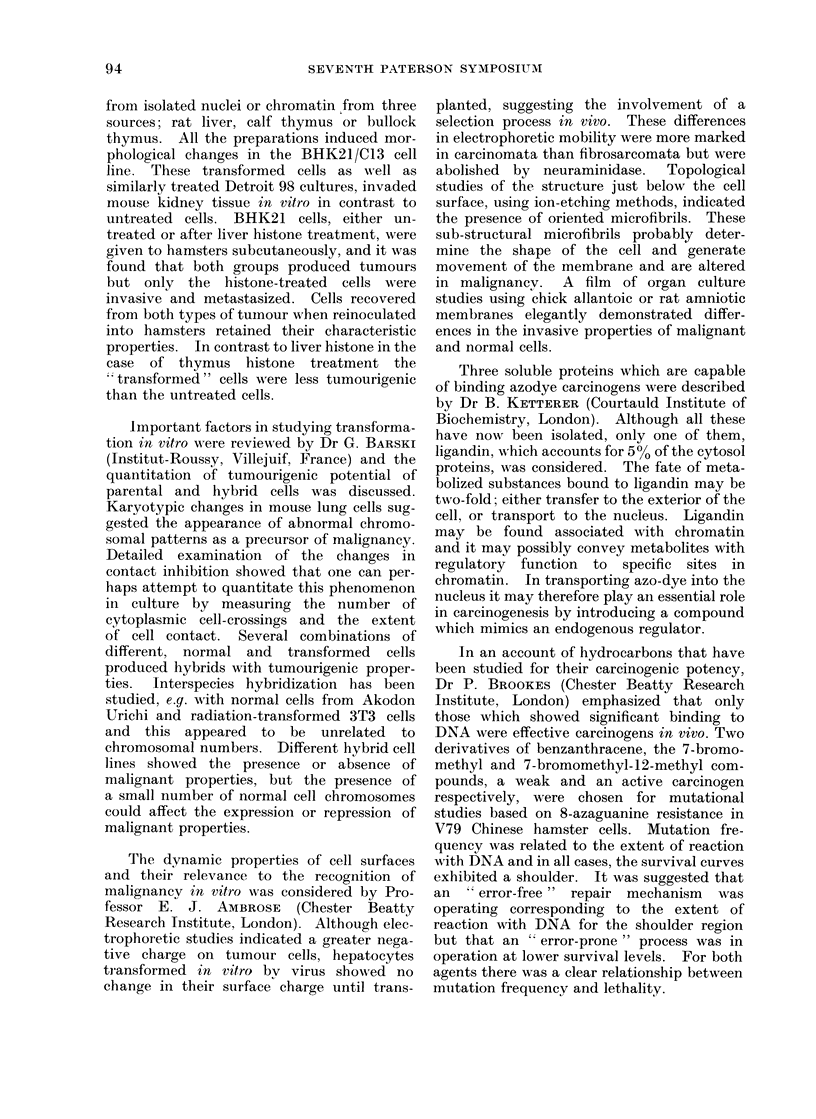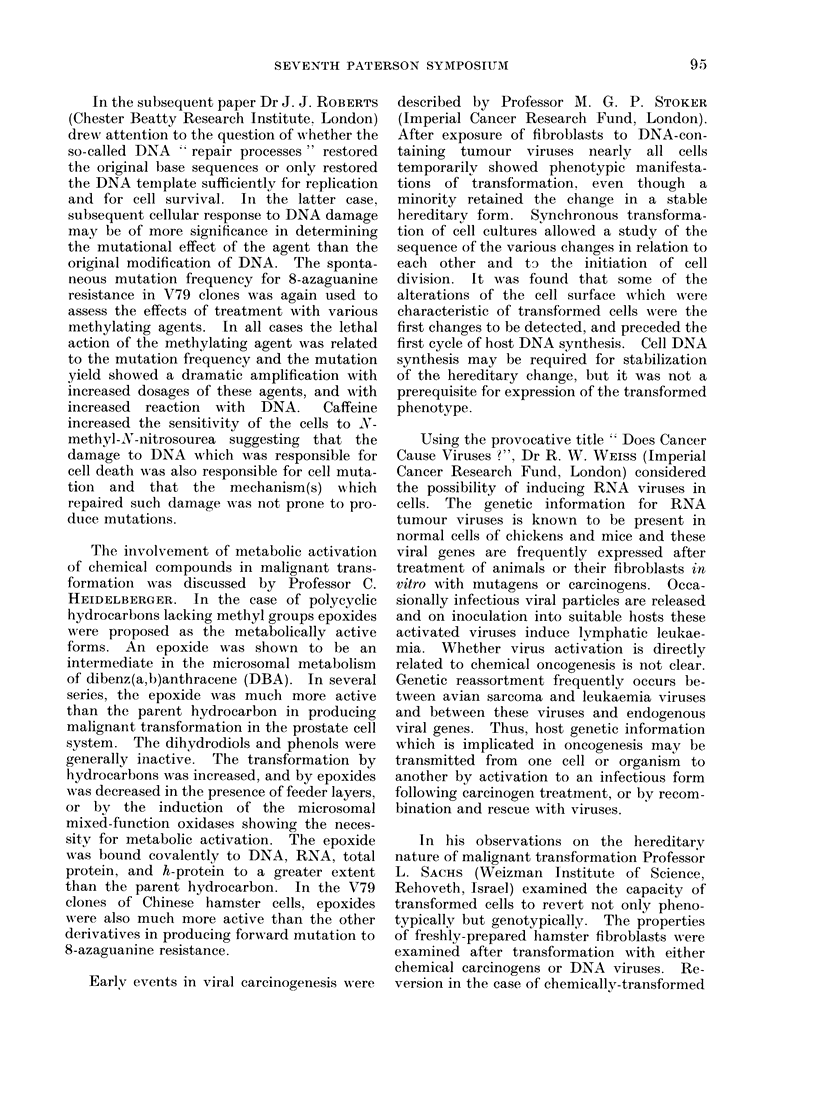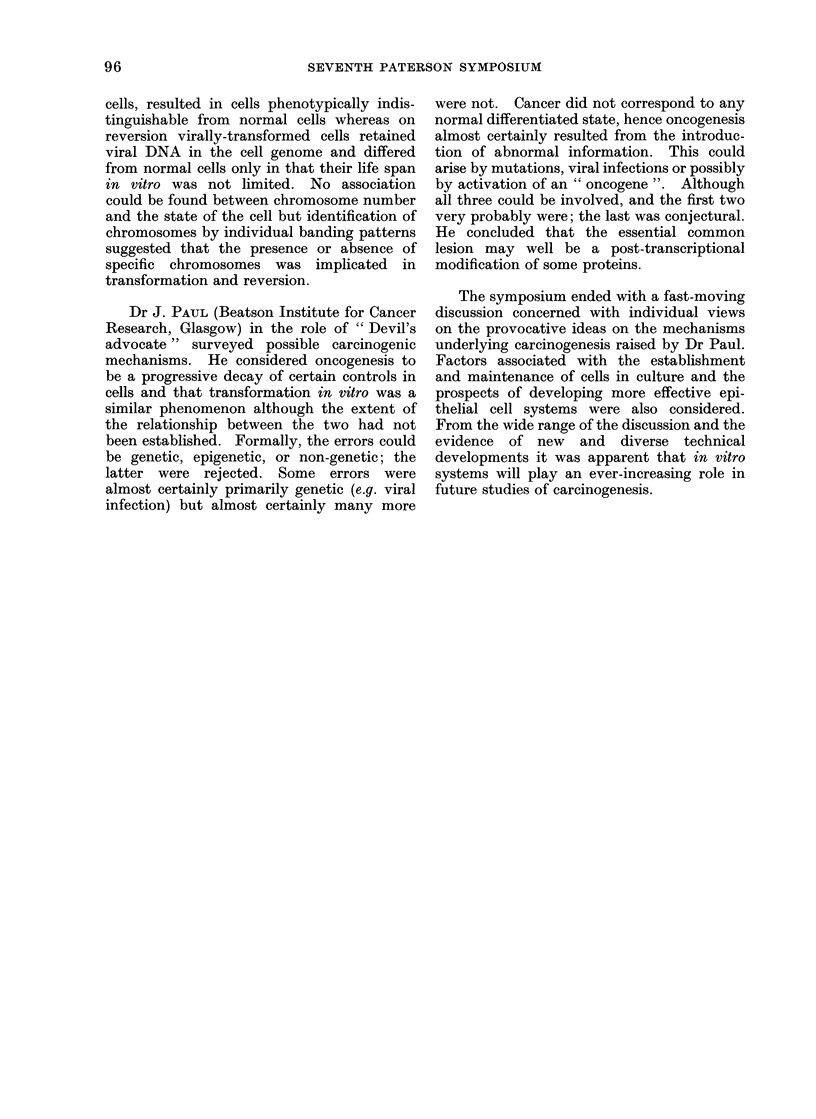# Seventh Paterson Symposium on Carcinogenesis in vitro

**Published:** 1973-01

**Authors:** 


*Br. J. Cancer* (1972) **26**, 92

### SEVENTH PATERSON SYMPOSIUM ON CARCINOGENESIS *IN VITRO*

Current interest in the possibility of carcinogenesis *in vitro* is motivated by the need for efficient and economical screening systems for environmental carcinogens—and equally, by the need for simpler systems than the whole animal for the study of molecular mechanisms involved in malignant transformation. The development of *in vitro* systems which have direct and proper relevance to the *in vivo* situation is therefore a highly desirable goal. While *in vitro* viral oncogenesis has reached an important stage in this respect, studies with chemical or physical carcinogens are still in their early phases of development. For these reasons, a small working symposium was held at the Paterson Laboratories, Christie Hospital and Holt Radium Institute, Manchester on October 1–4, 1972 under the title " Carcinogenesis *in vitro* " with the aim of evaluating the current status and trends of development in this field.

In the opening paper, Professor C. HEIDELBERGER (McArdle Laboratories, University of Wisconsin, Wisconsin, U.S.A.) stressed the need for systems of chemical carcinogenesis in culture in order to examine fundamental questions about involvement of cellular mechanisms. The major quantitative systems currently in use, fibroblast cell lines from adult C3H mouse ventral prostate, hamster embryonic fibroblasts and a new C3H/3T½ system, were described. Their relevance to carcinogenesis *in vivo* can be demonstrated, for example, by the presence of multiple, non-cross-reacting surface antigens in transformed clones derived from cloned parental non-transformed cells. The dependance of transformation on phases of the cell cycle system was also discussed.

Professor R. W. BALDWIN (Cancer Research Campaign Laboratories, University of Nottingham) reviewed the characteristics of neoantigens expressed on chemically-induced tumours. Tumour-specific rejection antigens, measured by the immune response elicited from transplanted tumour cells in syngeneic hosts, were present in many, but not all, chemically induced tumours and also on cells transformed *in vitro*. These antigens which were located specifically within the plasma membrane, were highly characteristic for individual tumours. Another class of neoantigens expressed on chemically-induced tumours were embryonic components detectable by reaction with lymphocytes or serum from multiparous donors. These antigens differed from the tumour-specific components in being common to several tumour types. Characterization of these antigens as well as other types, such as the products of carcinogen-binding to cellular macromolecules provided specific markers for the study of *in vitro* carcinogenesis.

Interactions *in vitro* of chemicals, virus, and radiation were examined by Dr J. A. DIPAOLO (National Cancer Institute, National Institutes of Health, Bethesda, U.S.A.). The ability of chemical carcinogens to induce transformation in Syrian hamster cells or Balb 3T3 was determined and quantitated. These transformed cells gave rise to cell lines which produced tumours when inoculated into appropriate hosts. Furthermore, the linear relationship of transformation and dose was consistent with the "one hit" hypothesis, *i.e.* this was an inducable rather than a selective phenomenon. Induction in the hamster cell system is believed to be direct as there is no indication of viral involvement. Evidence was also given of a relationship *in vitro* of structure and activity with numerous carcinogens that paralleled *in vivo* results. Different classes of chemical carcinogens are capable of producing transformation. X-irradiation, SV-7 virus, weak chemical carcinogens such as benzanthracene and enzyme inducing agents such as 7,8-benzflavone were all capable of enhancing transformation.

Dr P. T. IYPE (Paterson Labs, Christie Hospital and Holt Radium Institute, Manchester) described the properties of highly contact-inhibited liver cell lines from adult rats, which he had established in culture. They had round nuclei with two nucleoli, a low nuclear cytoplasmic ratio and a smooth surface. Many mitochondria and desmosomes were present—the latter emphasizing the epithelial nature of the cells. Liver specific, but not embryonic antigens were found on the cell surface. Finally, the cells did not grow in soft agar or produce tumours in rats. Antigenic changes, some of them cross-reacting, were found as early as 4 weeks after carcinogen treatment and before the cells had acquired the capacity to form colonies in soft agar.

Professor H. KATSUTA (Institute of Medical Sciences, University of Tokyo, Japan) also discussed changes observed in liver cells established in culture. Using a system where a zone of a sloping culture was washed intermittently by the medium (the " Nagisa " system) liver cells were found to grow more actively in this zone and to develop characteristics of transformed cells, although on transplantation they failed to form tumours. The electrophoretic mobility of the Nagisa-transformed cells was greater than normal and similar to that of malignant cells induced *in vivo*. However, pretreatment with sialidase prior to electrophoresis did not affect the Nagisa-transformed cells but substantially reduced the mobility of the malignant cells. Liver cells in monolayer culture treated with 4-nitroquinoline-1-oxide were transformed, but acquired no resistance to the chemical carcinogen on repeated treatment. In a time lapse film the only abnormal characteristic of the repeated treatment was rapid cellular movement; some of these cell-lines produced tumours on transplantation.

Dr G. PARMIANI (Istituto Nazionale per lo Studio e la Cure dei Tumori, Milan) reported on *in vitro* and *in vivo* studies of transformation in millipore-filter diffusion chambers. The cell-impermeable two-compartment diffusion chamber had an internal environment which was effectively protected from immunological action *in vivo*. Mouse fibroblasts in these diffusion chambers were exposed to methylcholanthrene (MC) for 4–24 weeks either in the peritoneal cavities of mice or *in vitro*. Fibrosarcomata developed on transplantation of the treated cells into immunodepressed syngeneic mice; the incidence was 60% for cells derived from *in vivo* systems and 50% from *in vitro* systems. No neoplastic transformation occurred in untreated diffusion chamber cultures. There was considerable variation in the immunogenicity of the transformed cells which could not be correlated with *in vitro* or *in vivo* origin, time of exposure, latency or growth rate.

Dr I. LASNITSKI (Strangeways Research Lab., Cambridge) discussed the effects of carcinogenic hydrocarbons and cigarette smoke condensates on epithelial tissues in organ culture. These included human and mouse lung, mouse trachea and skin, and mouse and rat prostate. In all tissues the components stimulated epithelial cell division leading to extensive hyperplasia. At the same time stromal growth was inhibited and the newly formed cells showed cytological changes compatible with malignant transformation. They became pleomorphic and in some tissues assumed invasive properties or underwent squamous metaplasia. Vitamin A suppressed the squamous changes, or if added after the carcinogen, reversed the hyperplasia. Testosterone, dihydrotestosterone and hydrocortisone reduced the effect of the carcinogen on the prostate gland while anti-androgens such as oestradiol-17$\beta$ and cyproterone acetate increased it.

The subject of transformation of epithelial cells in organ culture was also considered by Professor T. T. CROCKER (University of California, Irvine, California, U.S.A.). Studies were made of the action of carcinogens on tracheal organ cultures from suckling and adult hamster, and foetal, late foetal and adult monkey After treatment with benzpyrene the tissue became disordered and the columnar state of the tracheal surface became less differentiated together with an increased basal hyperplasia. The effect of vitamin A administered together with the carcinogen was to maintain a differentiated system although there was still hyperplastic activity similar to that induced by vitamin A alone. A study of carcinogenic and non-carcinogenic hydrocarbons showed a good correlation of their action *in vitro* with their carcinogenic activity *in vivo*. However, attempts to identify the changes in culture with carcinogenesis, by grafting treated mice trachea were unsuccessful.

The effects of histones on cells in culture were described by Professor A. L. LATNER (Cancer Research Unit, Royal Victoria Infirmary, Newcastle). Histones were prepared from isolated nuclei or chromatin from three sources; rat liver, calf thymus or bullock thymus. All the preparations induced morphological changes in the BHK21/C13 cell line. These transformed cells as well as similarly treated Detroit 98 cultures, invaded mouse kidney tissue *in vitro* in contrast to untreated cells. BHK21 cells, either untreated or after liver histone treatment, were given to hamsters subcutaneously, and it was found that both groups produced tumours but only the histone-treated cells were invasive and metastasized. Cells recovered from both types of tumour when reinoculated into hamsters retained their characteristic properties. In contrast to liver histone in the case of thymus histone treatment the "transformed" cells were less tumourigenic than the untreated cells.

Important factors in studying transformation *in vitro* were reviewed by Dr G. Barski (Institut-Roussy, Villejuif, France) and the quantitation of tumourigenic potential of parental and hybrid cells was discussed. Karyotypic changes in mouse lung cells suggested the appearance of abnormal chromosomal patterns as a precursor of malignancy. Detailed examination of the changes in contact inhibition showed that one can perhaps attempt to quantitate this phenomenon in culture by measuring the number of cytoplasmic cell-crossings and the extent of cell contact. Several combinations of different, normal and transformed cells produced hybrids with tumourigenic properties. Interspecies hybridization has been studied, *e.g.* with normal cells from Akodon Urichi and radiation-transformed 3T3 cells and this appeared to be unrelated to chromosomal numbers. Different hybrid cell lines showed the presence or absence of malignant properties, but the presence of a small number of normal cell chromosomes could affect the expression or repression of malignant properties.

The dynamic properties of cell surfaces and their relevance to the recognition of malignancy *in vitro* was considered by Professor E. J. Ambrose (Chester Beatty Research Institute, London). Although electrophoretic studies indicated a greater negative charge on tumour cells, hepatocytes transformed *in vitro* by virus showed no change in their surface charge until transplanted, suggesting the involvement of a selection process *in vivo*. These differences in electrophoretic mobility were more marked in carcinomata than fibrosarcomata but were abolished by neuraminidase. Topological studies of the structure just below the cell surface, using ion-etching methods, indicated the presence of oriented microfibrils. These sub-structural microfibrils probably determine the shape of the cell and generate movement of the membrane and are altered in malignancy. A film of organ culture studies using chick allantoic or rat amniotic membranes elegantly demonstrated differences in the invasive properties of malignant and normal cells.

Three soluble proteins which are capable of binding azodye carcinogens were described by Dr B. Ketterer (Courtauld Institute of Biochemistry, London). Although all these have now been isolated, only one of them, ligandin, which accounts for 5% of the cytosol proteins, was considered. The fate of metabolized substances bound to ligandin may be two-fold; either transfer to the exterior of the cell, or transport to the nucleus. Ligandin may be found associated with chromatin and it may possibly convey metabolites with regulatory function to specific sites in chromatin. In transporting azo-dye into the nucleus it may therefore play an essential role in carcinogenesis by introducing a compound which mimics an endogenous regulator.

In an account of hydrocarbons that have been studied for their carcinogenic potency, Dr P. Brookes (Chester Beatty Research Institute, London) emphasized that only those which showed significant binding to DNA were effective carcinogens *in vivo*. Two derivatives of benzanthracene, the 7-bromomethyl and 7-bromomethyl-12-methyl compounds, a weak and an active carcinogen respectively, were chosen for mutational studies based on 8-azaguanine resistance in V79 Chinese hamster cells. Mutation frequency was related to the extent of reaction with DNA and in all cases, the survival curves exhibited a shoulder. It was suggested that an "error-free" repair mechanism was operating corresponding to the extent of reaction with DNA for the shoulder region but that an "error-prone" process was in operation at lower survival levels. For both agents there was a clear relationship between mutation frequency and lethality.

In the subsequent paper Dr J. J. ROBERTS (Chester Beatty Research Institute, London) drew attention to the question of whether the so-called DNA " repair processes " restored the original base sequences or only restored the DNA template sufficiently for replication and for cell survival. In the latter case, subsequent cellular response to DNA damage may be of more significance in determining the mutational effect of the agent than the original modification of DNA. The spontaneous mutation frequency for 8-azaguanine resistance in V79 clones was again used to assess the effects of treatment with various methylating agents. In all cases the lethal action of the methylating agent was related to the mutation frequency and the mutation yield showed a dramatic amplification with increased dosages of these agents, and with increased reaction with DNA. Caffeine increased the sensitivity of the cells to *N*-methyl-*N*-nitrosourea suggesting that the damage to DNA which was responsible for cell death was also responsible for cell mutation and that the mechanism(s) which repaired such damage was not prone to produce mutations.

The involvement of metabolic activation of chemical compounds in malignant transformation was discussed by Professor C. HEIDELBERGER. In the case of polycyclic hydrocarbons lacking methyl groups epoxides were proposed as the metabolically active forms. An epoxide was shown to be an intermediate in the microsomal metabolism of dibenz(a,b)anthracene (DBA). In several series, the epoxide was much more active than the parent hydrocarbon in producing malignant transformation in the prostate cell system. The dihydrodiols and phenols were generally inactive. The transformation by hydrocarbons was increased, and by epoxides was decreased in the presence of feeder layers, or by the induction of the microsomal mixed-function oxidases showing the necessity for metabolic activation. The epoxide was bound covalently to DNA, RNA, total protein, and *h*-protein to a greater extent than the parent hydrocarbon. In the V79 clones of Chinese hamster cells, epoxides were also much more active than the other derivatives in producing forward mutation to 8-azaguanine resistance.

Early events in viral carcinogenesis were described by Professor M. G. P. STOKER (Imperial Cancer Research Fund, London). After exposure of fibroblasts to DNA-containing tumour viruses nearly all cells temporarily showed phenotypic manifestations of transformation, even though a minority retained the change in a stable hereditary form. Synchronous transformation of cell cultures allowed a study of the sequence of the various changes in relation to each other and to the initiation of cell division. It was found that some of the alterations of the cell surface which were characteristic of transformed cells were the first changes to be detected, and preceded the first cycle of host DNA synthesis. Cell DNA synthesis may be required for stabilization of the hereditary change, but it was not a prerequisite for expression of the transformed phenotype.

Using the provocative title " Does Cancer Cause Viruses ?", Dr R. W. WEISS (Imperial Cancer Research Fund, London) considered the possibility of inducing RNA viruses in cells. The genetic information for RNA tumour viruses is known to be present in normal cells of chickens and mice and these viral genes are frequently expressed after treatment of animals or their fibroblasts *in vitro* with mutagens or carcinogens. Occasionally infectious viral particles are released and on inoculation into suitable hosts these activated viruses induce lymphatic leukaemia. Whether virus activation is directly related to chemical oncogenesis is not clear. Genetic reassortment frequently occurs between avian sarcoma and leukaemia viruses and between these viruses and endogenous viral genes. Thus, host genetic information which is implicated in oncogenesis may be transmitted from one cell or organism to another by activation to an infectious form following carcinogen treatment, or by recombination and rescue with viruses.

In his observations on the hereditary nature of malignant transformation Professor L. SACHS (Weizman Institute of Science, Rehoveth, Israel) examined the capacity of transformed cells to revert not only phenotypically but genotypically. The properties of freshly-prepared hamster fibroblasts were examined after transformation with either chemical carcinogens or DNA viruses. Reversion in the case of chemically-transformed cells, resulted in cells phenotypically indistinguishable from normal cells whereas on reversion virally-transformed cells retained viral DNA in the cell genome and differed from normal cells only in that their life span *in vitro* was not limited. No association could be found between chromosome number and the state of the cell but identification of chromosomes by individual banding patterns suggested that the presence or absence of specific chromosomes was implicated in transformation and reversion.

Dr J. Paul (Beatson Institute for Cancer Research, Glasgow) in the role of " Devil's advocate " surveyed possible carcinogenic mechanisms. He considered oncogenesis to be a progressive decay of certain controls in cells and that transformation *in vitro* was a similar phenomenon although the extent of the relationship between the two had not been established. Formally, the errors could be genetic, epigenetic, or non-genetic; the latter were rejected. Some errors were almost certainly primarily genetic (*e.g.* viral infection) but almost certainly many more were not. Cancer did not correspond to any normal differentiated state, hence oncogenesis almost certainly resulted from the introduction of abnormal information. This could arise by mutations, viral infections or possibly by activation of an " oncogene ". Although all three could be involved, and the first two very probably were; the last was conjectural. He concluded that the essential common lesion may well be a post-transcriptional modification of some proteins.

The symposium ended with a fast-moving discussion concerned with individual views on the provocative ideas on the mechanisms underlying carcinogenesis raised by Dr Paul. Factors associated with the establishment and maintenance of cells in culture and the prospects of developing more effective epithelial cell systems were also considered. From the wide range of the discussion and the evidence of new and diverse technical developments it was apparent that *in vitro* systems will play an ever-increasing role in future studies of carcinogenesis.